# Increased Abundance of Achromobacter xylosoxidans and Bacillus cereus in Upper Airway Transcriptionally Active Microbiome of COVID-19 Mortality Patients Indicates Role of Co-Infections in Disease Severity and Outcome

**DOI:** 10.1128/spectrum.02311-21

**Published:** 2022-05-17

**Authors:** Priti Devi, Ranjeet Maurya, Priyanka Mehta, Uzma Shamim, Aanchal Yadav, Partha Chattopadhyay, Akshay Kanakan, Kriti Khare, Janani Srinivasa Vasudevan, Shweta Sahni, Pallavi Mishra, Akansha Tyagi, Sujeet Jha, Sandeep Budhiraja, Bansidhar Tarai, Rajesh Pandey

**Affiliations:** a INtegrative GENomics of HOst-PathogEn (INGEN-HOPE) Laboratory, CSIR-Institute of Genomics and Integrative Biology (CSIR-IGIB), Delhi, India; b Academy of Scientific and Innovative Research (AcSIR), Ghaziabad, India; c Max Super Speciality Hospital (A Unit of Devki Devi Foundation), Delhi, India; Nanchang University

**Keywords:** co-infection, pathogen genomics, host-pathogen interactions, COVID-19, disease sub-phenotype, nasopharyngeal RNA, respiratory virus oligo panel (RVOP), Holo-Seq, transcriptionally active microbial isolates, disease outcome, metabolic pathways

## Abstract

The modulators of severe COVID-19 have emerged as the most intriguing features of SARS-CoV-2 pathogenesis. This is especially true as we are encountering variants of concern (VOC) with increased transmissibility and vaccination breakthroughs. Microbial co-infections are being investigated as one of the crucial factors for exacerbation of disease severity and complications of COVID-19. A key question remains whether early transcriptionally active microbial signature/s in COVID-19 patients can provide a window for future disease severity susceptibility and outcome? Using complementary metagenomics sequencing approaches, respiratory virus oligo panel (RVOP) and Holo-seq, our study highlights the possible functional role of nasopharyngeal early resident transcriptionally active microbes in modulating disease severity, within recovered patients with sub-phenotypes (mild, moderate, severe) and mortality. The integrative analysis combines patients’ clinical parameters, SARS-CoV-2 phylogenetic analysis, microbial differential composition, and their functional role. The clinical sub-phenotypes analysis led to the identification of transcriptionally active bacterial species associated with disease severity. We found significant transcript abundance of Achromobacter xylosoxidans and Bacillus cereus in the mortality, Leptotrichia buccalis in the severe, Veillonella parvula in the moderate, and Actinomyces meyeri and *Halomonas* sp. in the mild COVID-19 patients. Additionally, the metabolic pathways, distinguishing the microbial functional signatures between the clinical sub-phenotypes, were also identified. We report a plausible mechanism wherein the increased transcriptionally active bacterial isolates might contribute to enhanced inflammatory response and co-infections that could modulate the disease severity in these groups. Current study provides an opportunity for potentially using these bacterial species for screening and identifying COVID-19 patient sub-groups with severe disease outcome and priority medical care.

**IMPORTANCE** COVID-19 is invariably a disease of diverse clinical manifestation, with multiple facets involved in modulating the progression and outcome. In this regard, we investigated the role of transcriptionally active microbial co-infections as possible modulators of disease pathology in hospital admitted SARS-CoV-2 infected patients. Specifically, can there be early nasopharyngeal microbial signatures indicative of prospective disease severity? Based on disease severity symptoms, the patients were segregated into clinical sub-phenotypes: mild, moderate, severe (recovered), and mortality. We identified significant presence of transcriptionally active isolates, Achromobacter xylosoxidans and Bacillus cereus in the mortality patients. Importantly, the bacterial species might contribute toward enhancing the inflammatory responses as well as reported to be resistant to common antibiotic therapy, which together hold potential to alter the disease severity and outcome.

## INTRODUCTION

The Coronavirus 2019 disease (COVID-19), caused by the severe acute respiratory syndrome coronavirus 2 (SARS-CoV-2), has led to unprecedented medical emergencies throughout the world, culminating into a pandemic. Since its first detection in Wuhan, China in 2019, the rate of infectivity and mortality due to SARS-CoV-2 has incessantly increased over time, with the present count reaching approximately 513 million cases including 6.2 million deaths globally (https://covid19.who.int/). An intriguing feature of COVID-19 is its remarkable heterogeneity in terms of clinical presentation, which has been a cause of concern and research throughout. For a significant fraction, COVID-19 presents as asymptomatic or mild self-limiting respiratory disease. Yet, in about 15% of the cases, the disease might escalate to progressive pneumonia with severe complications requiring intensive care or even lead to multiple organ failure and death (1–3). The precise determinants of disease severity are still confounding, limiting the scope for early medical intervention and disease management. Genomic alterations or mutations acquired by the SARS-CoV-2 virus during transmission is one of the factors. This aspect is vividly demonstrated during the emergence and perpetuation of new variants of SARS-CoV-2 in different populations with differential rates of infection, hospitalization and mortality (4, 5). Despite several reports showing association of mutations with mild, severe, and mortality outcomes (6, 7), yet, others have highlighted genomic similarities among the infecting SARS-CoV-2 strains, albeit with different clinical manifestations (8, 9). Thus, there has been effort toward understanding the role of host-related factors in disease severity. Advanced age, high basal metabolic index (BMI), male gender, immune related factors, and comorbidities are risk factors for mortality in COVID-19 patients (10–12). There still seems to be a missing link to explain, understand, and elucidate the diversity of disease severity and outcome.

In this background, it is important to evaluate the third factor for its functional role in disease severity and outcome. Although limited, important insights are emerging toward the plausible role of microbial community in susceptibility to COVID-19 (13, 14). The initial site of SARS-CoV-2 infection, the upper airways (UA) tract, houses bacterial, viral, and fungal populations (15) wherein the cross talk between the primary infecting pathogen and co-inhabiting microbes has been shown to modulate subsequent development of the disease with differential disease trajectory (16). SARS-CoV-2 infection may potentially alter the microbiota by pathogenic dominance or increasing levels of upper respiratory commensal bacteria, leading to disease progression and poor prognosis (17). Furthermore, the initial host-viral interactions in the nasal cavity and in the UA are crucial to modulate the subsequent systemic immune response to SARS-CoV-2 (18). Altered immune functions in response to co-infecting microbes, can increase the susceptibility of COVID-19 patients to severity (19) which is reflected in high rates of co-infection reaching up to 50% in non-survivors of COVID-19 (20).

These cues raise curiosity regarding the co-infecting microbes contributing toward differential disease severity in COVID-19, especially in severe and deceased patients. More importantly, can we identify *early microbial signatures/transcriptionally active isolates* for prioritizing patient sub-groups for priority health care? Presence of early resident microbe signatures would give a window for patient specific response during the progression of the disease. Thus, co-infections deserve closer attention to understand and evaluate their role in disease management and patient outcome (21). Few studies have illustrated the function of respiratory microbiome in SARS-CoV-2 infection, emphasizing the role of co-infecting pathogens as prospective modulators of COVID-19 (22–25).

The present study has explored and elucidated the early alterations in the respiratory microbiome, especially transcriptionally active isolates, in the patient cohort from India which could drive the COVID-19 disease severity sub-phenotypes. Through an integrative genomics approach, combining clinical data, SARS-CoV-2 genome information, and resident microbes, we have highlighted the significance of clinical parameters, SARS-CoV-2 *plus* respiratory viruses, transcriptionally active microbial diversity, their relative abundance, functional inferences, and enrichment of metabolic pathways of the nasopharyngeal microbiome in COVID-19 patients with different clinical sub-phenotypes and outcomes. Specifically, the identification of significant differential presence of transcriptionally active bacterial species in severe and mortality patients, with the metabolic pathway analysis, allowed us to propose a plausible mechanism that might help to understand additional aspects leading to COVID-19 severity.

## RESULTS

### Patient clinical characteristics and disease severity in COVID-19 subgroups.

**FIG 1:**
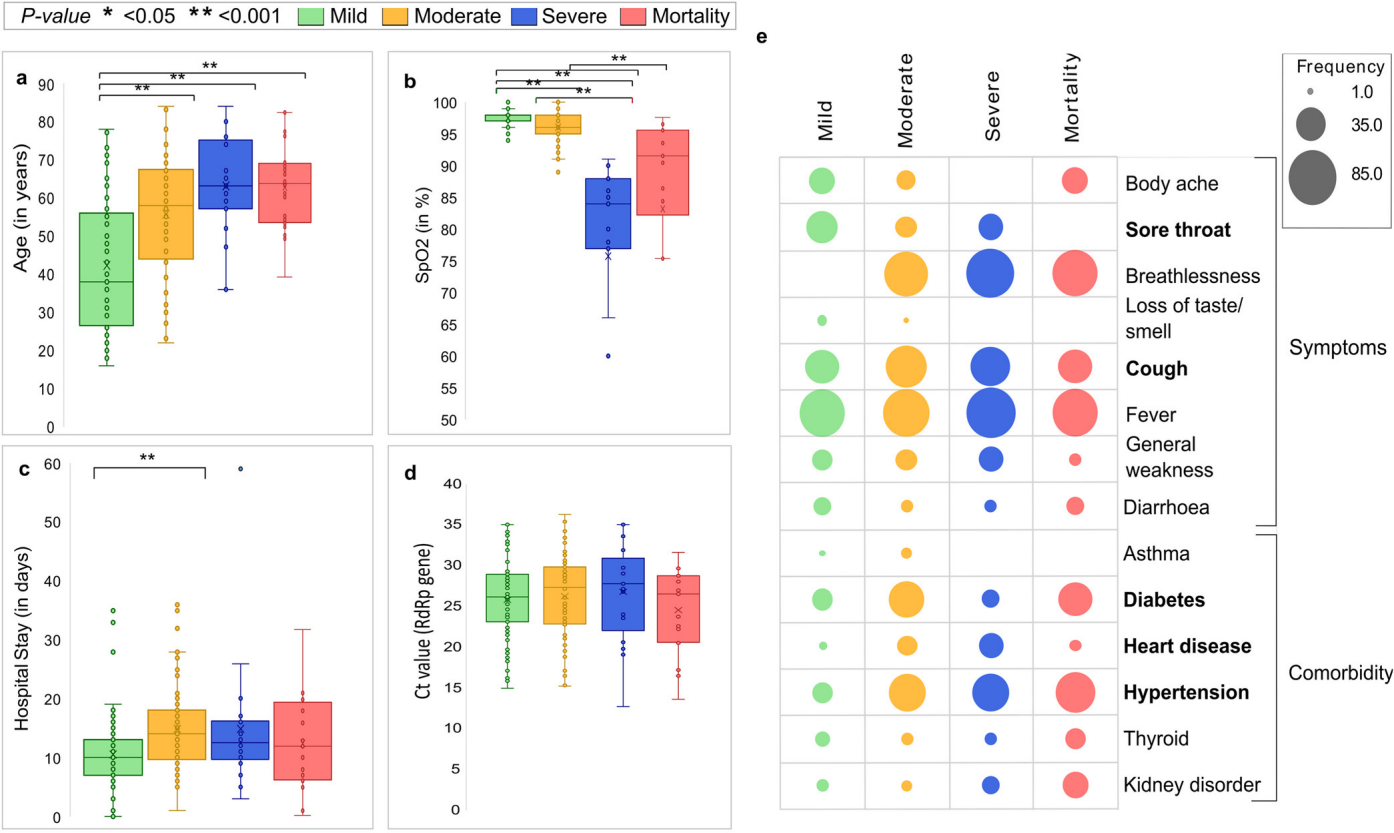
Representation of the clinical demographics of the COVID-19 patients. Continuous variables like (a) age, (b) SpO_2_, (c) hospital stay, (d) SARS-CoV-2 RT-PCR Ct value for RdRp gene in four disease severity sub-phenotypes: mild (green), moderate (yellow), severe (blue), and mortality (red), with statistical significance measured using Mann-Whitney *U* test. * represents *P*-value <0.05, ** represents *P*-value <0.001. (e) Categorical clinical features like symptoms and comorbidity information for the patients in the four severity sub-phenotypes: mild (green), moderate (yellow), severe (blue), and mortality (red).

A total of 198 COVID-19 patients included in the study were initially segregated into two broad categories based on clinical outcome: recovered (*n *= 177) and mortality (*n *= 21). Recovered patients were further divided into three sub-phenotypes: mild (*n *= 85; 42.9%), moderate (*n *= 73; 36.8%), and severe (*n *= 19; 9.6%). The clinical parameters were thoroughly investigated for plausible significant factors that could account for the difference in the disease severity and outcome among the patients Table 1. The statistical correlations and numerical patient distribution of continuous and categorical variables, respectively, across all patient categories has been illustrated in Fig. 1.

**TABLE 1:** Clinical characteristics of COVID-19 patients^*a*^

Groups	Mortality (*n *= 21)	Recovered (*n *= 177)	*P*- value	Mild (*n *= 85)	Moderate (*n *= 73)	Severe (*n *= 19)	*P*-value
Age	64.5 (54.75 to 69.25)	52 (32 to 65)	**0.0021** ^*b*^	38 (27 to 56)	58 (44 to 67)	63 (58 to 74.5)	**<0.001** ^*d*^
Gender (F|M)	4|16^*f*^	49|128	0.639^*c*^	32|53	11|62	6|13	**0.006** ^*c*^
Ct value							
E gene	25.25 (19.85 to 27.75)	25.11 (22.19 to 27.53)	0.378^*b*^	24.815 (22.5 to 27.23)	25.1 (21.64 to 27.7)	25.98 (22.82 to 28.73)	0.592^*d*^
RdRp	26.51 (21.39 to 28.46)	26.525 (23.11 to 29.40)	0.254^*b*^	26.145 (23.18 to 28.93)	27.12 (22.96 to 29.68)	29.04 (23.52 to 31.38)	0.487^*d*^
Signs and symptoms							
SpO^2^	92 (85.5 to 95.5)	97 (95 to 98)	**<0.001** ^*b*^	98 (97 to 98)	96 (95 to 98)	84 (77.5 to 88)	**<0.001** ^*d*^
Body ache	5 (23.80)	29 (15.93)	0.584^*c*^	20 (23.52)	9 (12.32)	0 (0)	0.108^*c*^^,^^*e*^
Sore throat	0 (0)	42 (23.07)		27 (31.76)	11 (15.06)	4 (21.05)	**0.046** ^*c*^
Breathlessness	14 (66.67)	63 (34.61)	**0.011** ^*c*^	0 (0)	48 (65.75)	15 (78.94)	0.409^*c*^^,^^*e*^
Loss of taste and smell	0 (0)	4 (2.19)		3 (3.52)	1 (1.36)	0 (0)	0.723^*c*^^,^^*e*^
Cough	8 (38.09)	84 (46.15)	0.560^*c*^	32 (37.64)	42 (57.53)	10 (52.63)	**0.039** ^*c*^
Fever	15 (71.4)	131 (71.97)	0.993^*c*^	61 (71.76)	54 (73.97)	16 (84.21)	0.535^*c*^
General weakness	1 (4.76)	27 (14.83)	0.330^*c*^	12 (14.11)	11 (15.06)	4 (21.05)	0.747^*c*^
Diarrhea	2 (9.52)	14 (7.69)	0.867^*c*^	9 (10.58)	4 (5.479)	1 (5.26)	0.446^*c*^
Asymptomatic	0 (0)	3 (1.64)		3 (3.52)	0 (0)	0 (0)	
Comorbidities							
Asthma	0 (0)	4 (2.19)		1 (1.17)	3 (4.10)	0 (0)	0.507^*c*^^,^^*e*^
Diabetes	8 (38.09)	46 (25.27)	0.358^*c*^	13 (15.29)	31 (42.46)	2 (10.52)	**<0.001** ^*c*^
Heart disease	1 (4.76)	16 (8.79)	0.802^*c*^	2 (2.35)	10 (13.69)	4 (21.05)	**0.007** ^*c*^
Hypertension	11 (52.38)	55 (30.21)	0.086^*c*^	13 (15.29)	33 (45.20)	9 (47.36)	**<0.001** ^*c*^
Thyroid	3 (14.28)	12 (6.59)	0.427^*c*^	7 (8.23)	4 (5.47)	1 (5.26)	0.759^*c*^
Kidney disorders	5 (23.80)	10 (5.49)	**0.011** ^*c*^	5 (5.88)	3 (4.10)	2 (10.52)	0.554^*c*^
No comorbidities	4 (19.04)	84 (47.45)	**0.01** ^*c*^	53 (62.35)	25 (34.24)	6 (31.57)	**<0.001** ^*c*^
Hospital stay (days)	12 (6.75 to 18.5)	12 (8 to 15.5)	0.95^*b*^	10 (7 to 13)	14 (10 to 18)	12.5 (10 to 15.5)	**<0.001** ^*d*^

a This table highlights the trend and statistical significance of clinical parameters across patient severity and outcome classes. The groups were mild, moderate, and severe in severity classification and mortality, recovered patients for outcome classification. Sub-clinical groups and major clinical parameters are mentioned in bold letters. Values of significance are highlighted in bold. Data are shown as median (IQR) or *n*(%).

b Mann Whitney U test.

c Chi^2^ test.

d Kruskal Wallis test.

e Test between two non-zero values.

f Missing data.

Based on the clinical data, a preponderance of male patients (72.72%) was observed in the cohort as a whole, which was true for the mild, moderate, and severe patients of the recovered group (*P*-value = 0.006) as well as mortality patients, as observed globally (26). The median age of patients within the recovered group (52 years) was significantly different from that in the mortality group (64.5 years) (*P*-value = 0.021). Classification of recovered patients also shows age as a significant modulator of severity wherein we observed age to be increasing with disease severity (*P*-value = 0.001). Importantly, the median age of severe patients is comparable to deceased patients (64.5, 63 years) indicating the role of other factors in disease outcome (Fig. 1a). Possibly not unexpected, low peripheral oxygen saturation (SpO_2_) levels and breathlessness, were significant features of the mortality group compared to the recovered (Fig. 1b). Above factors reflect the association of these parameters with disease severity, and importance of these parameters for severity classification.

The presence of comorbid conditions predisposes patients to an unfavorable clinical course. The comorbid conditions of hypertension (52.38%), diabetes (38.09%), and kidney disorder (23.8%) prevailed more in the mortality group than the recovered in our study, yet the association was statistically significant only for kidney disorder (*P*-value = 0.011). Stratification within the recovered group revealed significant associations with comorbid conditions of diabetes (*P*-value < 0.001), hypertension (*P*-value < 0.001), and heart disease (*P*-value = 0.007). Overall, only 19.04% of patients from the mortality group did not carry any comorbid condition while 41.24% patients in the recovered group had no comorbidities (*P*-value = 0.01). Apart from significantly lower hospital stays in the mild group (average 10 days) when compared with moderate group (average 14 days) (*P*-value <0.001), both hospital stay and the SARS-CoV-2 RT-PCR based cycle threshold (Ct) values for *RdRp* and *E* gene were found to be similar across patient categories (Fig. 1c, d and e).

It was important to ascertain whether clinical factors above had plausible effect on the abundance of transcriptionally active microbial isolates between the disease severity sub-phenotypes. Thus, we checked for the confounding effects of statistically significant clinical parameters in our study cohort. We performed a correlation analysis (using Pearson’s correlation and point-biserial correlation) between bacterial transcript read counts and the statistically significant parameters of age and comorbidities, respectively. We find that the r-score between age and cumulative bacterial transcript read count was −0.0077 with *P*-value 0.94 (using Pearson’s correlation), which is not statistically significant. A point-biserial correlation analysis between bacterial transcript read counts and presence and absence of comorbidities, gave r-score of 0.104 and *P*-value of 0.33 which again is statistically non-significant. Taking together the above observations, it reiterates that in the present study cohort, age and comorbidities are not significant confounders.

### Genomic characteristics of SARS-CoV-2 across the clinical sub-phenotypes.

**FIG 2:**
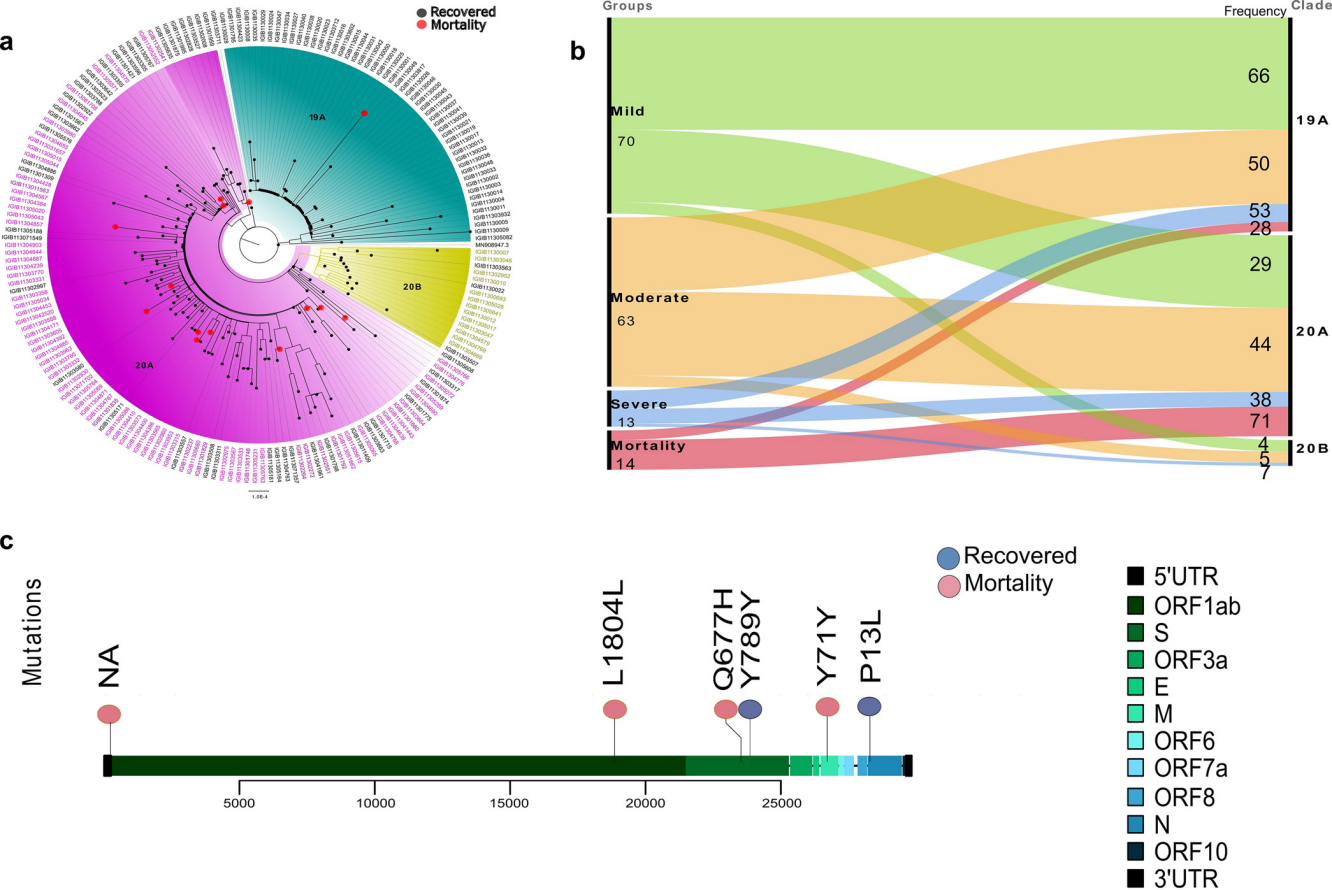
Mutation and phylogenetic study of the SARS-CoV-2 strains from COVID-19 patients. (a) Phylogenetic classification of SARS-CoV-2 genomes highlighted three clades: 19A, 20A, and 20B. The tip of the nodes (red) represents mortality patients whereas others represent recovered patients (black). (b) Distribution of samples from the four sub-phenotypes: mild, moderate, severe, mortality into different clades. (c) Lollipop plot displaying mutations having significant association with mortality (red) and recovered (blue) groups.

To observe the genomic characteristics of SARS-CoV-2, phylogenetic distribution and clades, as well as the mutational spectrum, were analyzed. Phylogenetic and clade distribution revealed that the majority of the samples belonged to the clades 19A (50.62%), and 20A (43.75%) with a relatively minor presence of clade 20B (5.62%) (Fig. 2a). Upon observing the presence of patient severity classes across SARS-CoV-2 clades, we note that 70% of the mortality cases were distributed in clade 20A but, every clade showed a substantial presence of other severity classes (Fig. 2b), thus, diluting the effect of clade specificity for any particular clinical sub-phenotype.

An in-depth analysis of the SARS-CoV-2 genomes at the mutation level allowed us to capture a total of 3,614 mutations across our sample cohort. Following the Fisher exact test, six mutations were found to be significantly linked with the clinical outcomes (*P*-value <0.05). A correlation analysis of these six mutations revealed association of two mutations, N:P13L (non-synonymous) and S:789Y (synonymous), with the recovered patients and four mutations, S:Q677H (non-synonymous) and ORF1b:1804L, 5’UTR:C241T, and M:71Y (synonymous), mutations with mortality patients (Fig. 2c; Table S1).

With few mutations identified to be significantly associated with the recovered and the mortality patients, further study is required to understand its functional importance in delineating disease severity and outcome, which merits an independent study. Thus, we hypothesized whether the nasopharyngeal transcriptionally active microbial isolates can provide predictive modulators of disease severity in the background of primary SARS-CoV-2 infection.

### Nasopharyngeal bacterial profiling and characterization across patient sub-phenotypes.

**FIG 3:**
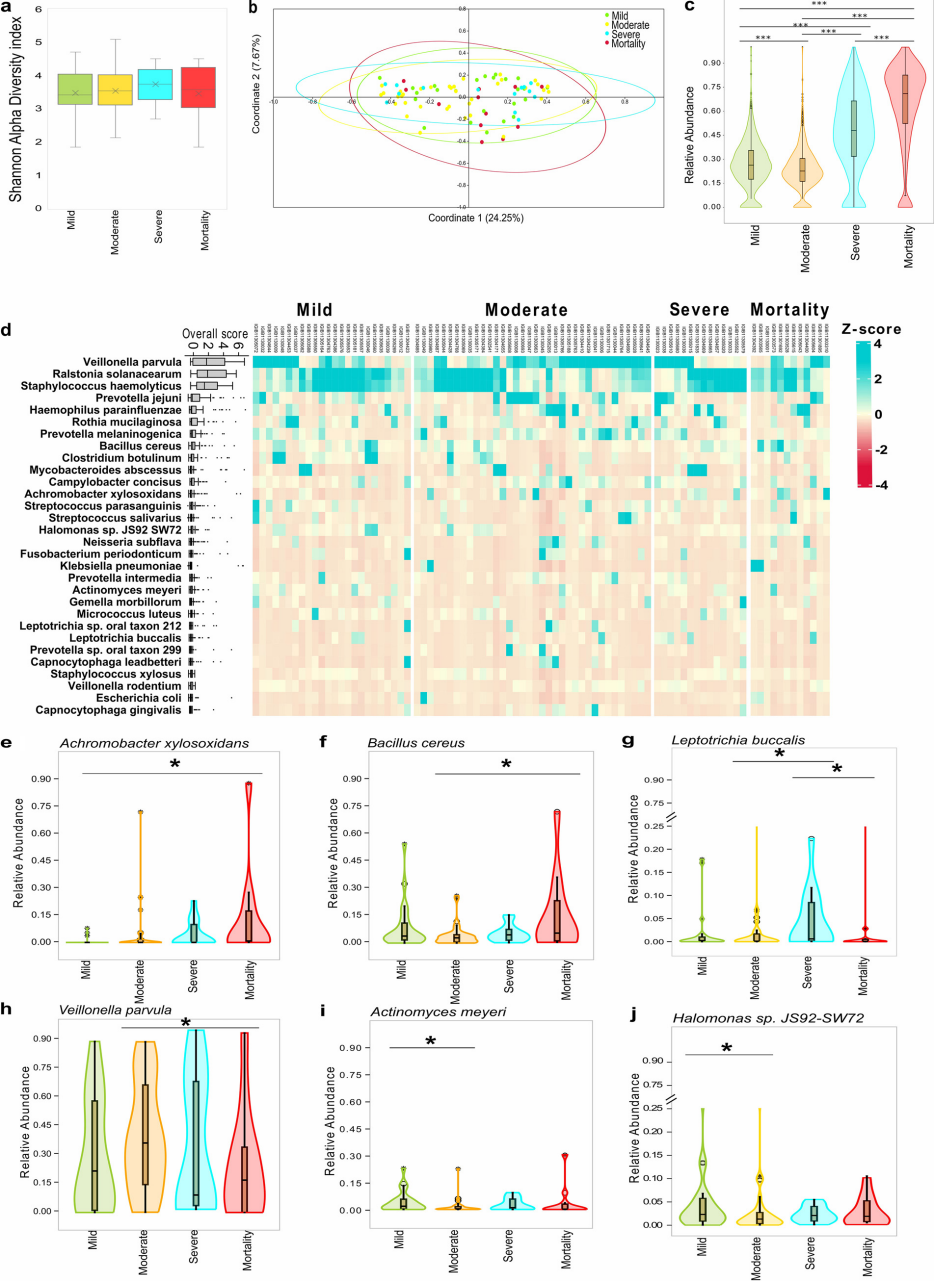
Bacterial species diversity and abundance profile between four sub-phenotypes from Holo-transcriptome study. (a) Shannon alpha diversity index of bacterial species. (b) Principal coordinate analysis (PCoA) plot representing the beta diversity of bacterial species using Bray-Curtis dissimilarity matrix. (c) Total bacterial reads abundance based on transcriptional active isolates between four sub-groups, significance calculated using Kruskal Wallis test. The pairwise significance was calculated using Mann-Whitney U test. *** represents a *P*-value < 0.001. (d) The heatmap represents the distribution of top 30 bacterial species in sub-groups. The boxplot on the left represents the overall transcriptional active isolates of the bacterial species in the cohort. (e to j) Significant differentially abundant bacterial species between mortality and recovered sub-groups (mild, moderate and severe), significance calculated using Mann-Whitney U test. * represents *P*-value <0.05. (e) Achromobacter xylosoxidans in mortality versus mild, (f) Bacillus cereus in mortality versus moderate, (g) Leptotrichia buccalis in severe versus moderate/mortality, (h) Veillonella parvula in moderate versus mortality, (i) Actinomyces meyeri, and (j) *Halomonas* sp. in mild versus moderate.

We used the holo-transcriptome analysis to understand the transcriptionally active microbial populations in a subset of patients with mild (*n *= 24), moderate (*n *= 36), severe (*n *= 14), and mortality (*n *= 12). Alpha diversity (Shannon index) values across all four sub-phenotypes was observed to be low with no significant difference between the effective number of species (ENS) across sub-phenotypes (Fig. 3a; Table S2), whereas Beta diversity (Bray-Curtis distance matrix) analysis (Fig. 3b) showed non-distinct clustering by PERMANOVA test. Interestingly, microbial abundance analysis via examining the transcriptionally active isolates revealed significant differences in the transcriptional intensity of bacterial species between the four sub-phenotypes wherein we specifically observed an enhanced presence of bacterial transcripts in severe and mortality patient’s nasopharynx compared with mild/moderate groups (Fig. 3c; Table S3). This finding led us to elucidate further as to which species were causing this differential abundance. Towards this, top 30 bacterial species were identified by mapping the relative bacterial transcriptional active isolates across all the 86 patients (Fig. 3d). Veillonella Parvula, Ralstonia solanacearum, Staphylococcus haemolyticus, and *Prevatolla Jejuni* were found to be highly abundant across all the four sub-phenotypes. An extensive literature review of top 30 bacterial species along with its reported association with SARS-CoV-2 has been provided as Table S4.

Each of the top 30 bacterial species were analyzed for its significant presence in any of the four clinical sub-phenotypes. Importantly, we identified six bacterial species, Achromobacter xylosoxidans, Bacillus cereus, Leptotrichia buccalis, Veillonella parvula, Actinomyces meyeri, and *Halomonas* sp. showing significant differential transcriptional intensity across sub-phenotypes (Fig. 3e to j). Among these species, Achromobacter xylosoxidans and Bacillus cereus were significantly associated with mortality (Fig. 3e and f) whereas Leptotrichia buccalis with severe group (Fig. 3g). Although Veillonella parvula was found to be highly abundant across the entire cohort, a statistically significant differential enrichment of this species was observed in the moderate patients (Fig. 3h). Actinomyces meyeri and *Halomonas* sp. showed association with the mild compared to moderate (Fig. 3i and j). Identification of severity associated transcriptionally active microbes in COVID-19 have multiple potential applications, with future studies potentially unravelling the mechanistic association of these microbes with COVID-19 severity. To seed these future investigations, we categorized the bacterial metabolic pathways across COVID-19 sub-phenotypes.

### Metabolic pathways as a function of microbial diversity between clinical sub-phenotypes.

**FIG 4:**
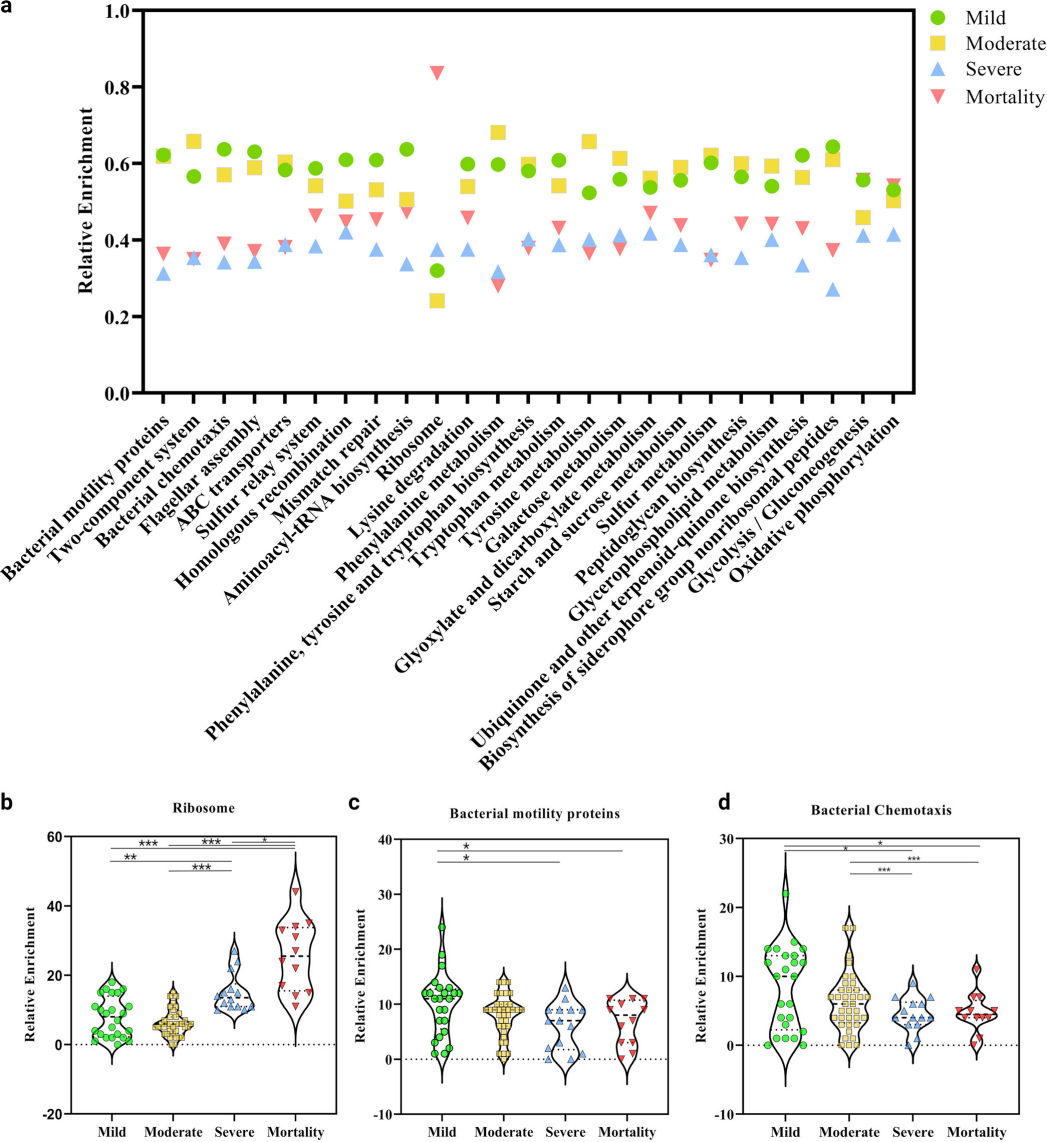
Relative enrichment of bacterial metabolic pathways across four clinical sub-phenotypes of COVID-19 patients. (a) Top 25 KEGG defined bacterial pathways highlighting cumulative distribution of significantly enriched bacterial metabolic pathways. Specific significant pathway differences between disease sub-phenotypes, (b) ribosome proteins, (c) bacterial motility proteins, and (d) bacterial chemotaxis. Statistical significance measured using Mann-Whitney U test. * represents *P*-value <0.05; ** represents *P*-value <0.01; *** represents *P*-value <0.001.

The extensive non-redundant catalogue of microbial genes (KEGG pathway analysis) identified differentially enriched pathways involved in bacterial functions (Table S5). The top selected pathways showing differential enrichment across the clinical sub-groups is depicted in Fig. 4a. The metabolic potential as revealed by carbohydrate and amino acid metabolism pathways showed depletion in severe and mortality patients as compared with mild and moderate. The metabolism and biosynthesis pathways of nearly all amino acids (particularly phenylalanine, tryptophan, tyrosine, and lysine) were decreased in abundance. Other carbohydrate metabolic pathways (galactose, glyoxylate and decarboxylate, and sucrose metabolic pathways) were consistently under-expressed in both severe and mortality. Thus, the bacterial competence to produce and metabolize nutrients seems to diminish with increase in clinical severity index in COVID-19 cases. Alternatively, glycolysis/gluconeogenesis and oxidative phosphorylation pathways in the mortality group showed expression similar to the mild group. Genetic information processing pathways: replication and repair (homologous recombination and mismatch repair) and folding, sorting and degradation (sulfur relay system) along with ABC transporters (membrane transport for sugars, metals, peptides, amino acids, and other metabolites) were relatively depleted in severe and mortality groups, suggesting altered adaptation to adverse microenvironment. On the other hand, the glycerophospholipid metabolism pathway was observed to be enriched in mild and moderate cases, possibly indicative of concerted efforts for bacterial adaptation to the microenvironment in these groups (27). Of note, the mortality and severe patients showed significant overexpression of ribosomal proteins (Fig. 4b) whereas, bacterial motility proteins and chemotaxis were significantly diminished (Fig. 4c, d). Ribosomal proteins might be an indicator of changes in growth rate as evident by more transcriptional intensity of rRNA in the microbiome of severe and mortality patients. Several studies highlight the correlation between rRNA abundance with the active proliferation of microbes inclusive of use of cellular rRNA as an indicator of *in situ* growth rate in various naturally occurring bacterial populations. Studies in Escherichia coli and Salmonella *typhimurium* have revealed that cellular RNA concentration is closely linked with growth rate (28–30). However, such correlation is not linear and there are evidences toward the inverse relationship between rRNA and active growth of microbes limiting the association between rRNA and growth rates (31–36).

The comparative analysis of enriched KEGG orthology identifiers (KO) terms against their corresponding metabolic pathways (Fig. 4a) revealed differential abundance across the sub-clinical phenotypes as shown in Table S6.

### Co-presence of respiratory viruses across COVID-19 patients.

**FIG 5:**
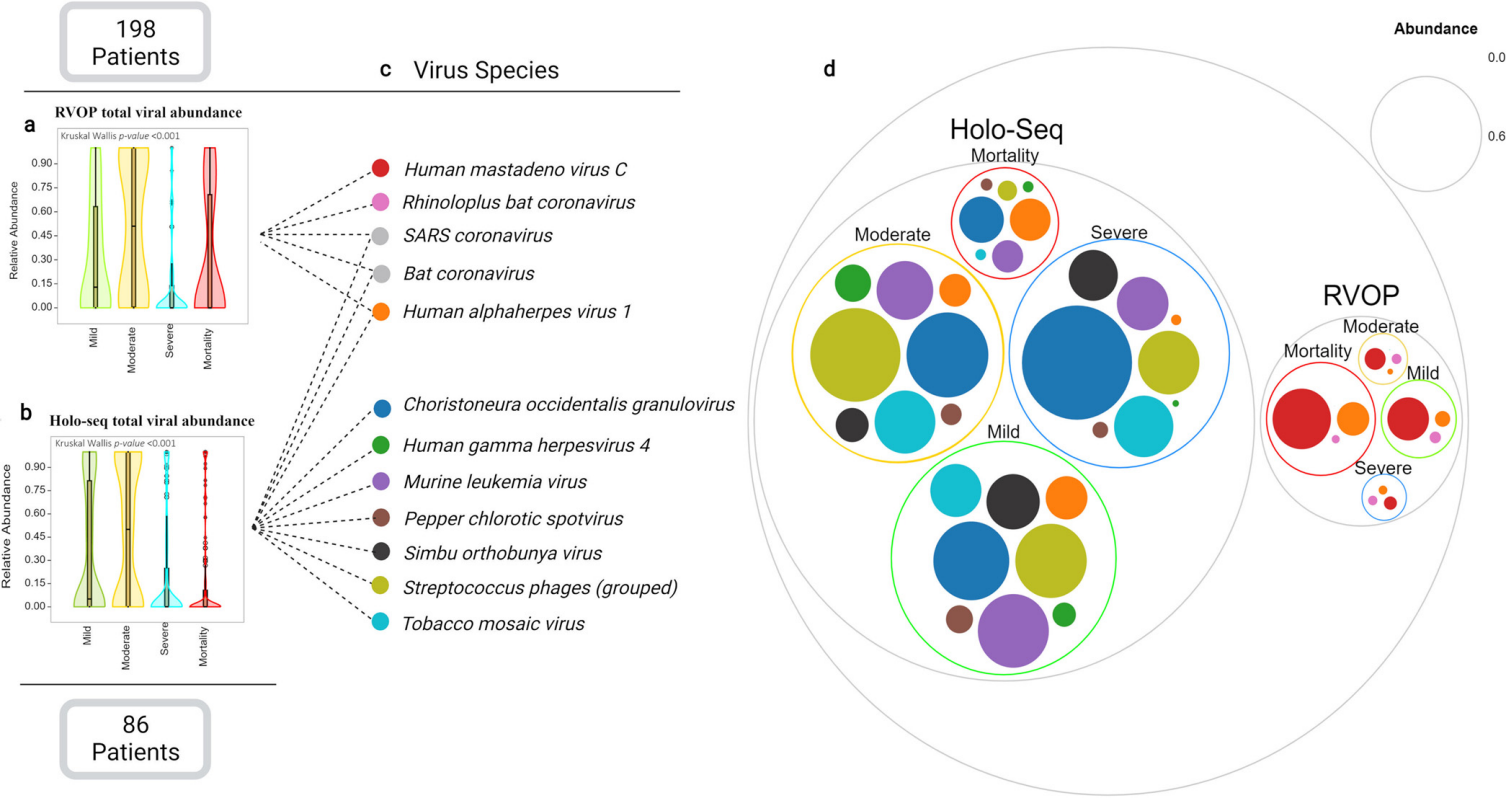
Viral species abundance from Holo-seq and RVOP across four clinical sub-phenotypes of COVID-19 patients. Total viral abundance captured using, (a) RVOP method, (b) Holo-seq method, (c) captured viral species with a relative abundance >1%, and (d) the Venn diagram represents the overall abundance of each viral species across four groups in both RVOP and Holo-seq.

Using RVOP and holo-transcriptome analysis, both methods identified significant differences in the abundance of viruses in the four sub-phenotypes with relatively higher viral abundance observed in mild and moderate patients (Fig. 5a, b; Table S4). A total of 12 different viruses were captured as illustrated in Fig. 5c. Significant differences in viral abundance was observed only for *Choristoneura occidentalis granulovirus* and *Tobacco mosaic virus*, however, we were unable to find association with the disease sub-phenotypes. We also note diminution of Streptococcus *Phages* and complete absence of *Simbu orthobunyavirus* from the mortality group (Fig. 5d). This suggests the depletion of viral diversity in the mortality group when considering the entire population of viral species.

## DISCUSSION

Disease severity in COVID-19 is orchestrated through a series of variables: viral, host, and resident microbes, leading to diversity of symptoms and an increase in the odds of mortality. Stratification of patients in different classes of severity can be a definitive approach to detangle the concomitant risk factors. This study utilizes an integrative approach involving different perspectives of host clinical characteristics, viral genome variations, and a comprehensive exploration of the nasopharyngeal microbiome threaded together, to elucidate the disease severity observed in the COVID-19 patient sub-phenotypes.

The clinical characteristics of the COVID-19 patients could only partly account for the severe and mortality group cases. The non-modifiable risk factors like age, gender, and comorbidity were in sync with previous studies which have shown detrimental effect during COVID-19 (37), yet, a complete association is difficult to comprehend. It is estimated that roughly 50% of the hospitalized patients had no reported comorbidity (38). Similarly, the clade diversity of the viral isolates revealed a proportional presence of mild, moderate, and severe patients in all the three clades—19A, 20A, and 20B. The dominant presence of mortality cases in clade 20A could be attributable to the fact that clade 20A became the source clade for VOCs (variants of concern) like Delta, Kappa, and Beta strains of SARS-CoV-2 (39). However, presence of mild and moderate patients in clade 20A points toward other plausible factors modulating the clinical outcome. Moreover, N:P13L mutation associated with the recovered group was identified in VUI-NP13L (variant of interest) in Southeast Brazil around June 2020 and was reported with low mortality rates and cases per million (40).

Q677H mutation associated with the mortality group emerged independently across six lineages in the United States and showed evidence of adaptation, due to its effect on the proximal polybasic furin cleavage site in the Spike protein (41). Importantly, the clinical impact of this mutation needs to be fully determined (42). Recently, it was reported to help increase the infectivity and confer neutralizing antibody resistance, particularly in background of other VoCs (43). The presence of these mutations in our cohort highlights the importance of genomics surveillance of the viral population but at the same time *in vitro* studies toward elucidating the mechanism/s is awaited.

The variable host clinical characteristics and the limited viral genetic diversity led us to explore whether alterations in the microbiome composition due to SARS-CoV-2 infection align with the scale of disease severity observed in our patient cohort. If yes, can such signatures be identified during the early phase of the infection with value for disease stratification? The composition of the nasal microbiome has been observed to be altered in several respiratory infections including COVID-19 affecting the course of the disease and clinical outcome (44). Different metagenomic studies portrayed decrease in the nasopharyngeal microbiome diversity in SARS-CoV-2 infected patients, leading to predominance of a specific microbe that correlated with symptom severity (22, 23, 45).

Although the present study did not reveal significant shifts in diversity and composition of the nasopharyngeal transcriptionally active isolates among the four clinical sub-phenotypes of COVID-19, this could be attributable to the Anna Karenina principle (AKP) of microbiome dysbiosis (46–48), which implies that a greater observed variability exists among individuals with dysbiotic microbiome. Additionally, multiple studies investigating the bacterial communities and the respiratory microbiome in COVID-19 revealed drastic reduction in diversity and composition with increasing disease severity (23, 49). Another plausible explanation for the observed differences relies on the transcriptomic profiling method utilized for the current study, which may capture bioactivity from a diverse community more effectively than the absolute DNA quantification methods commonly used for microbiome analyses (50, 51). Moreover, meta-transcriptome sequencing can capture the microbiome profiles at high resolution along with the active functional elements, which has been demonstrated to change (microbial gene expression) without large alterations in overall community structure (52).

Notably, a significant increase in the relative bacterial abundance (based on transcriptional activity) in the severe and mortality groups was observed when compared with the mild and moderate. This feature indicates the probable role of dysbiotic microbiome in disease severity. Majorly, dysbiosis is associated with alteration in the abundance of bacterial population causing opportunistic microbes to flourish or making way for pathogens for invasion (53, 54). Separate studies have identified enrichment of different species like *L. buccalis*, *V. parvula*, *C. gingivalis*, *P. melaninogenica*, *H. parainfluenzae*, *R. mucilaginosa*, and *N. subflava* in the oral microbial communities of COVID-19 patients (23, 55, 56). Moreover, different mechanisms have been suggested by which a dysbiotic nasopharyngeal microbiome, leading to an overgrowth of certain microbial species, can alter or cause progression in disease severity of COVID-19. A proposed mechanism is enrichment and subsequent migration of nasopharyngeal microbial species into the lungs, resulting in pneumonia and emphysema conditions (57). This proposition was also seen in different studies, where oral pathogens propagated a new disease when it migrated to other organs (23, 58, 59). Alternatively, a shift in the healthy microbial community due to disease conditions might alter the cytokine production and lead to an increase of both inflammatory response and clinical severity in respiratory diseases (60–62).

**FIG 6:**
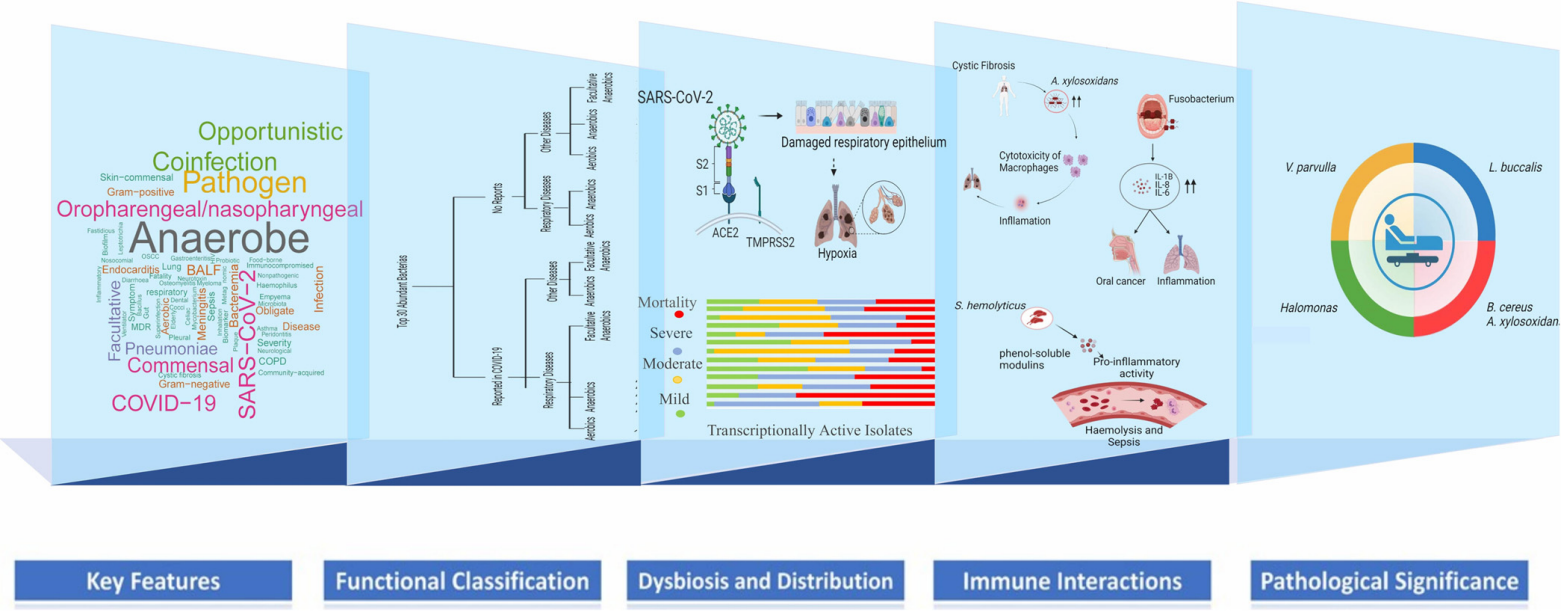
Key features and functional classification of the top 30 bacterial species across COVID-19 sub-phenotypes. A layered projection highlighting SARS-CoV-2 infection associated transcriptionally active microbiome dysbiosis leading to differential distribution of bacterial species in the sub-phenotypes; immunopathology of nasopharyngeal microbial species during dysbiosis; significantly abundant bacteria with their pathological correlation with disease severity.

Taken together, viral-host resident transcriptionally active microbes’ cross talk might initiate a sequence of dynamic events where the SARS-CoV-2 viral infection may modulate differential abundance of particular bacterial species, leading to *microbiome dysbiosis*. A similar interaction was shown by Susi et al., that competition for limited host resources may result in a “tragedy of the commons” situation, where non-optimal levels of host exploitation may emerge (63). These bacterial species might in turn aggravate the viral/primary infection through different mechanisms based on their inherent functional properties (Fig. 6).

Higher transcriptional intensity of Achromobacter xylosoxidans *and* Bacillus cereus in the mortality group may have implications in disease severity. A. xylosoxidans is an aerobic, motile, Gram negative bacteria that carries intrinsic as well as acquired mechanisms of resistance, conferring multidrug-resistance (MDR) phenotype (64). A. xylosoxidans, has emerged as an opportunistic pathogen, causing pulmonary infection in the cases of dysfunctional immune response or predisposing conditions like end stage renal disease or cardiac disease (65, 66). Interestingly, a study by Jabbar et al. reported A. xylosoxidans as the second most prevalent bacterial species found in severe COVID-19 patients with resistance to several classes of antibiotics (67). This bacteria is reported to enhance inflammation by increasing the production of cytokines such as IL-6, TNF-α, and G-CSF as seen in the case of cystic fibrosis (CF) (68).

Meanwhile, B. cereus, the facultative anaerobe enriched in mortality cases is known to cause infection via consumption of contaminated food and nosocomial transmission (69). Mainly, intestinal diseases such as diarrhea are reported to be caused by B. cereus yet, rarely though, B. cereus cause lower respiratory tract infections. Shimoyama et al. highlighted a case where the patient succumbed to lung infiltrates associated with B. cereus (70). A recent study shows that during COVID-19, B. cereus co-infection can be observed in a patient with probable immunocompromised state due to inhalation-based steroid use (71). Leptotrichia buccalis is an opportunistic pathogen commonly found in the oral microbiota and reported to cause severe pneumoniae in SARS-CoV-2-infected elderly individuals (72, 73). Similarly, *L. buccalis* was significantly enriched in the severe group of our cohort with a median age above 60 years. The transcriptional abundance of different microbial species between severe and mortality groups with similar age presentation of ~64 years suggests that the observed microbial differences in the study is a function of the disease severity. Interestingly, the other anaerobic opportunistic microbe, Veillonella parvula, has been reported as a marker for COVID-19 when compared with flu and healthy controls. In our patient cohort, *V. parvula* was found significantly enriched in the moderate group, although its substantial presence was detected across the cohort. Additionally, studies deciphered that both *V. parvula* and SARS-CoV-2 stimulate production of proinflammatory cytokines, mainly TNF-a, that might aggravate the inflammatory and pro-oxidative responses leading to diverse respiratory infection outcomes (74–77).

**FIG 7:**
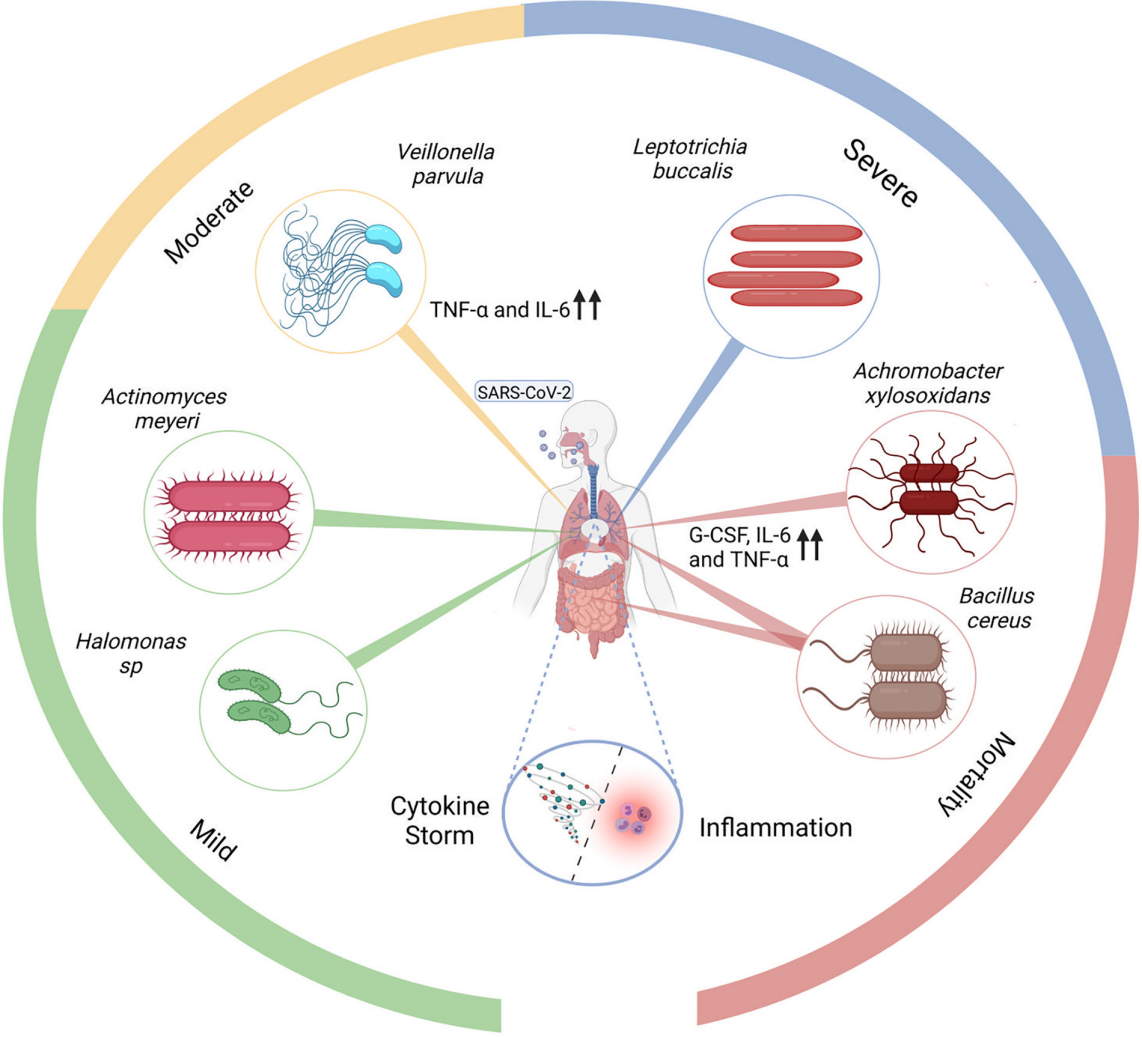
Possible functional role of specific transcriptionally active bacterial species in COVID-19 disease severity and outcome. Highlights the possible mechanism where enhanced presence of certain transcriptionally active microbes can aggravate the inflammatory cytokine response leading to disease severity.

On the other hand, Actinomyces meyeri and *Halomonas* spp. were reportedly enhanced in mild COVID-19 symptom patients. Disease association with *Halomonas* species is rarely reported (78) whereas *A. meyeri* is known to cause pneumonia and has a predilection for dissemination, yet reported cases have mild presentations (79). The significant abundance of specific microbial species across COVID-19 disease sub-phenotypes suggests their modulatory role in disease outcome. We summarized the current findings in Fig. 7.

The causal effect of the presence of different transcriptionally active microbial species in different classes of severity was carefully looked through the treatment regime for each of the subgroups. Based on the symptoms/symptom severity, Table 2 highlights treatment regime given to each of the subgroups. The administration of antibiotics to each sub-phenotype could have been beneficial in alleviating the symptoms and relief to patients, especially for the recovered group of patients. The presence of transcriptionally active isolates of different microbial species might be playing a modulatory role in disease severity, as exemplified by the presence of Achromobacter xylosoxidans and Bacillus cereus in the mortality group, where A. xylosoxidans is reportedly resistant to several classes of antibiotics (67). High transcriptionally active isolate abundance of A. xylosoxidans in the nasal microbiome in our mortality patients might predispose the host to severe respiratory viral infection and non-responsiveness to antibiotics. Although, it is pertinent to mention that we detected 2,417 genes for A. xylosoxidans with more than 80% similarity in our study, we did not find high confidence MDR A. xylosoxidans genes in the mortality patients. One of the probable reasons might be the time point of the transcriptional profiling, during which the MDR genes may not be actively expressed. Similarly, the resistance of B. cereus to penicillins and cephalosporins as a result of beta lactamase production is also reported to be leading cause of mortality in infected patients (80).

**TABLE 2:** Outline of the treatment regime given to the different sub-phenotypes of COVID-19 patients during hospital stay^*a*^

Groups	Mortality (*n *= 21)	Recovered (*n *= 177)	*P*- value	Mild (*n *= 85)	Moderate (*n *= 73)	Severe (*n *= 19)	*P*-value
Treatment						
Antimalarial	4 (19.04)	66 (36.26)	0.015^*b*^	34 (40)	25 (34.24)	7 (36.84)	0.756^*b*^
Antibacterial	3 (14.28)	21 (11.53)	0.974^*b*^	8 (9.41)	7 (9.58)	6 (31.57)	**0.019^*b*^**
Antiviral	4 (19.04)	65 (35.71)	0.172^*b*^	25 (29.41)	29 (39.72)	11 (57.89)	0.052^*b*^
Antibiotic	9 (42.85)	97 (53.29)	0.420^*b*^	46 (54.11)	42 (57.53)	9 (47.36)	0.718^*b*^
Antifungal	2 (9.52)	4 (2.19)	0.244^*b*^	2 (2.35)	1 (1.36)	1 (5.26)	0.785^*b*^
Anti-inflammatory	3 (14.28)	5 (2.74)	0.052^*b*^	3 (3.52)	2 (2.73)	0 (0)	0.862^*b*^^,^^*c*^
Anticoagulant	6 (28.57)	20 (10.98)	0.060^*b*^	7 (8.23)	7 (9.58)	6 (31.57)	**0.012^*b*^**

a Data are shown as median as *n*(%) with the Chi^2^ test applied for significance. Values of significance are highlighted in bold.

b Chi^2^ test.

c Test between two non-zero values.

Moreover, not only did the microbiomes demonstrate shifts in bacterial abundance and populations across the sub-phenotypes of COVID-19, but the dysbiosis was also functionally evident. The overall downregulation of metabolic pathways in severe and mortality groups reflected a reduction in microbial functions, pointing toward an unlikely impact on the host. The depletion of bacterial pathways associated with membrane transport (two component system and ABC transporters), bacterial chemotaxis, and cell motility in severe and mortality groups indicated toward lower bacterial potential for sensing and adapting to the environment which is consistent with a previous study of COVID-19 (23, 81, 82).

Additionally, altered amino acids pathways across severe and mortality groups’ microbiome could reportedly affect immune responses aggravating COVID-19 severity (83). Furthermore, the nasopharyngeal transcriptionally active microbiome of the mortality group patients did not show depletion of glycolytic pathway, wherein the micro-environment favors aerobic glycolysis (evident through growth of aerobic bacteria), a condition that also sustains high SARS-CoV-2 replication (84). Reasonably, we infer that nasopharyngeal microbiome dysbiosis during SARS-CoV-2 infection might enhance pathogen invasion and alter immune responses, contributing toward the observed clinical severity in different groups of COVID-19 patients. In parallel with the transcriptionally active bacterial microbiome profile, our work also characterized the local virome to understand the effect of SARS-CoV-2 infection at the primary site of entry. Studies have reported an increase in pathogenic viral species co-infecting the oral microbiome, accelerating disease severity (13). Yet, a significant difference in abundance and diversity of viral species among the four clinical sub-phenotypes was not observed, pointing toward possible viral interference mechanism due to SARS-CoV-2 infection (85, 86). Moreover, a reduction in phage population of the microbiome can be an effect that provides an additional milieu to opportunistic pathogens of the microbiome to grow and cause secondary infections accelerating the clinical course.

### Conclusion.

**FIG 8:**
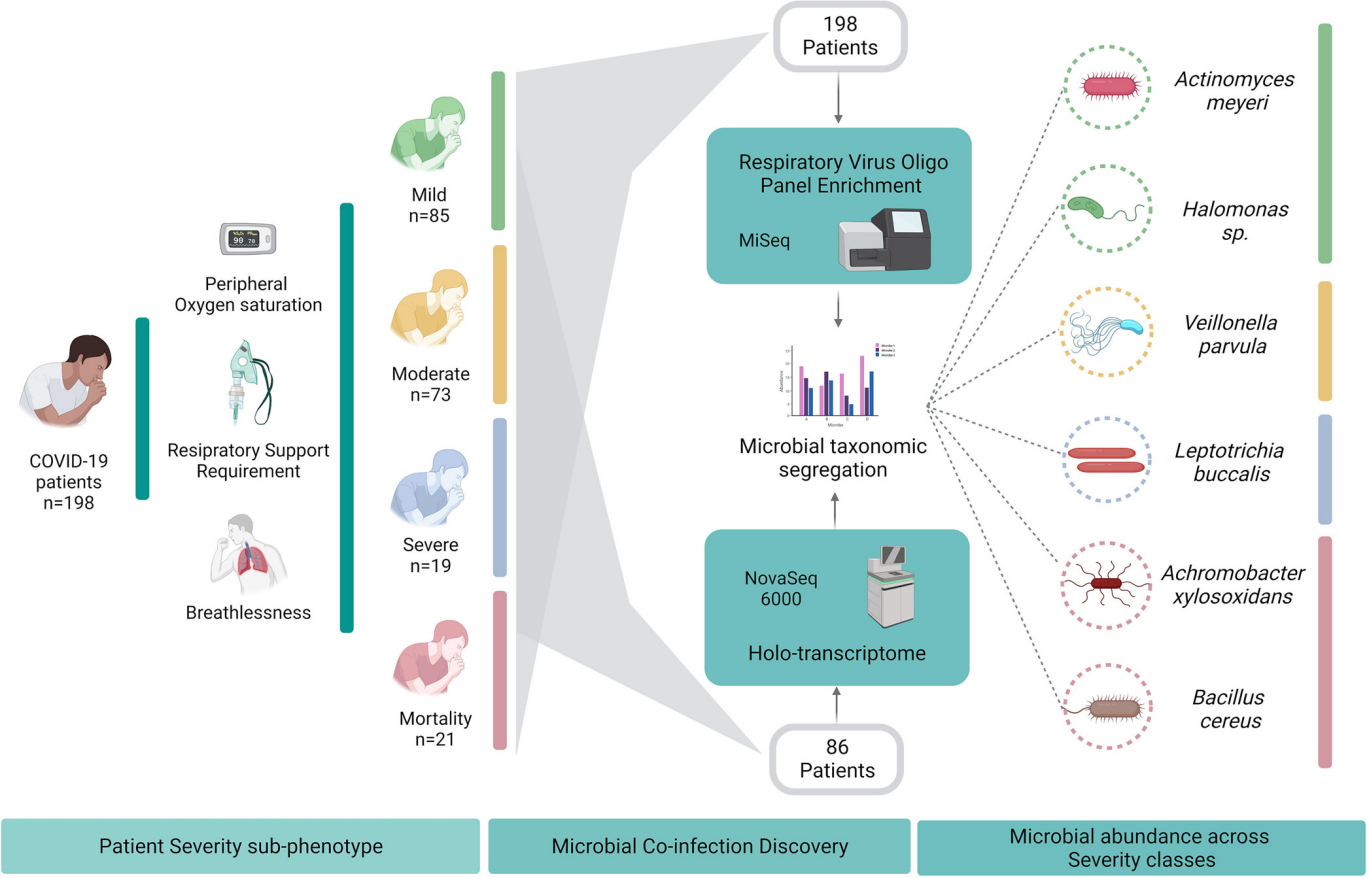
It highlights the study design and significant findings wherein microbial signatures for COVID-19 disease severity has been discovered in our hospital admitted cohort of patients.

The findings in the study offer an opportunity to bring forth the less explored modulatory role of the microbiome alterations and disease severity in a hospitalized cohort of COVID-19 patients from the Indian sub-continent Fig. 8. The differentially active isolates of certain bacterial species associated with clinical groups provide leads for evaluating their probable roles in modulating the disease course in COVID-19. This especially comes into play when it has been recognized that, for co-infecting bacterial species, differences exist between populations at risk, pathogen distribution, and antibiotic susceptibility. Future strategies can include exploring the microbial spectrum in COVID-19 patients from different geographical regions which might be beneficial in health care management.

The study can be strengthened in the future with the inclusion of the longitudinal sampling of the patients included in the study. That may enhance the scope to understand the dynamics of transcriptionally active microbial population change during the course of the disease as well as the functional dynamics. A more balanced gender representation could have been useful. However, because these are hospital-admitted patients during the COVID-19 disease, we appreciate the practical limitation. If possible, more than one hospital cohort could help expand the understanding and relevance of the findings made in the study.

## MATERIALS AND METHODS

### Design of the study.

The study was conducted with 198 COVID-19 patients enrolled between April 2020 to July 2020, to characterize the abundance of transcriptionally active isolates of different co-infecting bacteria and viruses inhabiting the nasopharyngeal cavity of COVID-19 patients. Detailed clinical presentation and demographic data along with RT-PCR test results and disease outcomes from each patient’s electronic medical record were collected and carefully documented for its usage during analysis.

### Sample collection and preprocessing.

The patients were admitted to the MAX Hospital, Delhi, India with confirmed COVID-19 positive status based on RT-PCR results. The nasopharyngeal and/or throat swabs were collected in viral transport media (VTM) solution by the paramedical staff at the hospital on the day of reporting, by trained medical staff with required safety precautions inclusive of PPE, face mask, and gloves.

### Clinical subgrouping of study participants.

The patients were categorized into four sub-phenotypes based on disease severity and outcome: mild, moderate, severe, and mortality, as per Indian Council of Medical Research (ICMR) guidelines (Comprehensive Guidelines for Management of COVID-19 patients, Directorate General of Health Services, MoHFW, GOI). Briefly, SpO_2_ levels, requirement of respiratory support, and/or breathlessness parameters were taken into consideration. In mild cases, the SpO_2_ level was ≥ 94% with no breathing problem. Moderate patients were defined as showing breathing difficulty with SpO_2_ levels ranging between 91% and 93%. Severe patients showed respiratory distress with respiratory support requirement and SpO_2_ levels < 90%. Mortality group was defined as patients who succumbed to COVID-19 during hospital stay. Mild, moderate, and severe were clubbed together into one group as “recovered” compared with the mortality cases, for some of the analysis included in the manuscript.

### Viral RNA isolation and qRT-PCR.

Viral RNA from VTM solutions was isolated using QIAmp viral minikit, Qiagen, Cat. No. 52906 and SARS-CoV-2 detection and quantification was performed using TRUPCR SARS-CoV-2 kit (3B BlackBio Biotech India Ltd., Cat. No. 3B304) with a cycle threshold of 35.

### Library preparation and sequencing.

Whole genome sequencing of the 198 RT-PCR positive samples, using the capture based Illumina Respiratory Virus Oligo Panel (RVOP), was done to capture SARS-CoV-2 genome as well as additional co-presence of other respiratory viruses. Using a combination of clinical data, disease sub-phenotype, and availability of RNA, a subset of 86 samples of the 198 total samples were studied to explore the presence of transcriptionally active microbes using Holo-Seq (Holo-transcriptome). The library preparation protocols for RVOP and Holo-transcriptome have been previously published from our lab (87). Briefly, double stranded cDNA was prepared from 100 ng RNA using Superscript IV first-strand synthesis system (Thermo Fisher Scientific, Cat. No. 18091050) and DNA polymerase I Large (Klenow) Fragment (New England Biolabs, Cat. No. M0210S). The RVOP library was prepared using Illumina DNA Prep with Enrichment kit (Illumina, Cat. No. 20018705) and the Holo-transcriptome library was prepared using Illumina TruSeq Stranded Total RNA Library Prep Gold (Illumina, Cat. No. 20020598) as per the manufacturer’s protocol. Agilent 2100 bioanalyzer was used to check the quality of both libraries. The RVOP library was denatured and diluted to optimal loading concentration for sequencing on MiSeq platform, using v3 reagent kit at 2 × 75 bp read length. The Holo-transcriptome library was sequenced on the NovaSeq 6000 system, using the NovaSeq SP reagents v1 at 2 × 101 read length at 400pM loading concentration.

### Sequencing data analysis and metatranscriptomic analysis.

The sequencing data analysis was performed as previously published from our lab (87). FastQC v0.11.9 was used to check the Phred quality score for all sequences (Babraham Bioinformatics, 2020a – FastQC A Quality Control tool for High Throughput Sequence Data). The quality score threshold was 20 and above. Adapter trimming was performed using the TrimGalore v0.6.6 and alignment of sequences was performed using the HISAT2 v2.2.1 algorithm on human data build hg38 (88, 89). SAM tools v1.12 were used to remove aligned human sequences (90). Henceforth, only unaligned sequences were taken into consideration. BCFTools v1.12. generated consensus FASTA and variant calling, which was followed by the alignment of sequences to the 40 respiratory virus panel of Illumina RVOP, to explore the presence of respiratory viruses in addition to SARS-CoV-2 (91). The detected species were counted using the number of reads mapped per species. Kraken2 was used to assign taxonomic labels to microbial species detected from the RVOP and the Holo-Seq analysis (92). The output from the metagenomic classification of the detected species obtained from Kraken was analyzed further using the Pavian software (93).

### *De novo* assembly and pathway enrichment analysis.

We used MEGAHIT v1.2.9 (94) to perform the *de novo* assembly of the samples using the raw sequencing reads, and contigs larger than 150 bp were retained to predict the genes by MetaGeneMark v3.25 (95) using default parameters. Then, CD-HIT v4.8.1 was applied for gene clustering and merging each sample. Finally, redundant sequences with sequence similarity and alignment lengths above 95% of the sequence length were removed. The functional profiles were annotated according to Kyoto Encyclopedia of Genes and Genomes (KEGG) orthology (http://www.genome.jp/kegg/) with the maximum e-value cut-off 1*10 − 5, minimum identity of 95%, and minimum alignment length of 15 amino acids for proteins.

### Mutational and phylogenetic study.

Of 198 samples, 160 SARS-CoV-2 genomes (with >50% genome coverage) were used for phylogenetic analysis, as previously described by Mehta et al. (7). Clade assignment to all the genomes was done using Nextclade (https://clades.nextstrain.org/). The vcf files were used for mutational analysis. We applied a nonparametric Fisher exact test of significance (for independence between two categorical variables) on our mutation data set which consisted of the total set of mutations identified for the study cohort (independent of its presence in recovered or mortality). *P*-values were calculated from two-sided tests using 0.05 as the significance level. The direction of the association between the mutation and group (recovered/mortality) was calculated using phi-coefficient correlation (r_φ_) by measuring the strength of association, henceforth identifying the significant mutations for the mortality as well as recovered patients. Gviz and trackViewer packages from R were used to plot the lollipop plot to visualize the mutations (96, 97).

### Statistical analysis.

The data was described using descriptive statistics, which display continuous variables as medians or interquartile ranges and categorical variables as percentages or proportions. Wherever appropriate, we compared the differences using the ANOVA, Mann–Whitney *U* test and Chi square testing. To compare the distribution of bacterial presence across our patient categories, we employed the Kruskal Wallis test. The Shannon Diversity index (H) was calculated to characterize the bacterial species diversity in patient samples used for the Holo-transcriptomics study to account for the abundance and evenness of bacterial species in each patient sample. For analysis of beta diversity, we performed principal coordinate analysis (PCoA) in PAST software using Bray-Curtis dissimilarity matrix and PERMANOVA was calculated to determine the statistical significance of beta diversity (https://palaeo-electronica.org/2001_1/past/issue1_01.htm).

### Data availability.

The data sets presented in this study can be found online at the NCBI-SRA under the accession numbers PRJNA676016 and PRJNA678831 the consensus fasta are available at the GISAID-EpiCoV (https://www.gisaid.org/) under the submission IDs: EPI_ISL_5316892- EPI_ISL_5317001 and EPI_ISL_5317004 - EPI_ISL_5317014.
